# A Sarcoma at the Site of Previous Extravasation of Adriamycin

**DOI:** 10.1080/1357714021000066386

**Published:** 2002-12

**Authors:** Joris Ceulemans, Ivo De Wever, Raf Sciot, Maria Debiec-Rychter, Allan T. van Oosterom

**Affiliations:** 1 Department of Surgical Oncology University Hospital Catholic University Leuven Herestraat 49 Leuven B-3000 Belgium; 2 Department of Pathology University Hospital Catholic University Leuven Herestraat Leuven B-3000 Belgium; 3 Centre for Human Genetics University Hospital Catholic University Leuven Herestraat Leuven B-3000 Belgium; 4 Department of Oncology University Hospital Catholic University Leuven Herestraat Leuven B-3000 Belgium

## Abstract

We report the case of a 66-year-old man presenting with a high-grade pleomorphic sarcoma at the left elbow 16 years after the extravasation of adriamycin given for a malignant ifbrous histiocytoma of the tibia.We suggest that this sarcoma originated in a multistep way over many years, out of the chronic inflammatory tissue that developed due to a non-specific cellular damage at the nuclear level, interfering with normal cell replication necessary for normal healing tissue healing. As a result, the non-healed chronic inflammatory tissue transformed over several years into a preneoplastic mesenchymal tumour and later into a high-grade pleomorphic sarcoma.


*Sarcoma* (2002) **6,** 135–139

Taylor & Francis healthsciences

**CASE REPORT**

### A sarcoma at the site of previous extravasation of adriamycin

JORIS CEULEMANS[1], IVO DE WEVER[1], RAF SCIOT[2], MARIA DEBIEC-RYCHTER[3] & ALLAN T. VAN OOSTEROM[4]

[1]*Department of Surgical Oncology,* [2]*Department of Pathology,* [3]*Centre for Human Genetics and* [4]*Department of Oncology, University Hospital Catholic University Leuven, Herestraat 49, B-3000 Leuven, Belgium*

**Abstract**
We report the case of a 66-year-old man presenting with a high-grade pleomorphic sarcoma at the left elbow 16 years after the extravasation of adriamycin given for a malignant fibrous histiocytoma of the tibia. We suggest that this sarcoma originated in a multistep way over many years, out of the chronic inflammatory tissue that developed due to a non-specific cellular damage at the nuclear level, interfering with normal cell replication necessary for normal healing tissue healing. As a result, the non-healed chronic inflammatory tissue transformed over several years into a preneoplastic mesenchymal tumour and later into a high-grade pleomorphic sarcoma.

**Key words:** *sarcoma, adriamycin, extravasation*

### Case report

A 66-year-old man was treated for a malignant fibrous histiocytoma of the proximal left tibia, without metastases, 16 years previously.

After one course of neoadjuvant chemotherapy with bleomycin–dactinomycin–cyclophosphamide and two cycles of high-dose methotrexate, an above-knee amputation of the left lower limb was performed.

Postoperatively the patient received adjuvant chemotherapy with CDDP–adriamycin (doxorubicin)–bleomycin–cyclophosphamide and dactinomycin.

During one of the chemotherapy administrations, extravasation of adriamycin occurred in the tissues around the basilic vein at the left elbow. No skin necrosis occurred. After a period of local erythema and swelling, a multinodular sclerotic lesion developed, consisting of four to five noduli of 0.5–1 cm diameter, fixed to the skin and underlying biceps muscle which also became indurated.

The lesions were situated along the basilic vein from the elbow up to where the adriamycin extravasation had occurred (Fig. 1).

Over a period of 16 years there was no evidence of tumour recurrence. The patient remained in good health and the nodules at his left elbow did not change in size or number. The patient consulted this year because of a fast growing mass above the left elbow at the extravasation site. One of the nodules had significantly increased in size over a period of 1 month.

On examination, the mass was 6 cm in diameter, fixed to the underlying biceps muscle and overlying skin, multinodular and non-tender.

Magnetic resonance imaging showed a tumour of $2 \times 2.5 \times 4$ cm on the ulnar side of the biceps muscle with thickening of the overlying skin (Fig. 2). Computerized tomography of chest and abdomen did not show metastases except for a small nodular lesion at the pleura of the left upper lobe.

A resection of the mass *en bloc* with the medial head of the biceps, part of the brachialis muscle, and the subcutaneous tissue and overlying skin was performed. A split thickness skin graft was used to cover the defect.

Postoperative wound healing was somewhat delayed because of partial loss of the skin graft due to a hematoma.

Two months after the procedure a new CT scan of the chest showed a clear increase in volume of the previously described nodule. Even more pleural nodules were seen, which were highly suggestive for pleural metastasis. The patient was subsequently treated with four cycles of ifosfamide with partial response: only the initially described nodule was still present. Via a left thoracotomy, a resection of the nodule *en bloc* with the lateral side of ribs 4 and 5 was done. A second nodule was palpated intraoperatively and resected with the lateral side of rib 3. Multiple scars in the left lung, especially in the lingula, were seen and one of these lesions was excised. A recon-

*Correspondence to:* I. De Wever, M.D. Ph.D., Department of Surgical Oncology, University Hospital Gasthuisberg, Herestraat 49, 3000 Leuven, Belgium. Tel: +32-16-34-68-31; Fax: +32-16-34-68-34; E-mail: Ivo.Dewever@uz.kuleuven.ac.be

ISSN 1357–714X print/ISSN 1369–1643 online/02/0200135–05 © Taylor & Francis Ltd
DOI: 10.1080/1357714021000066386

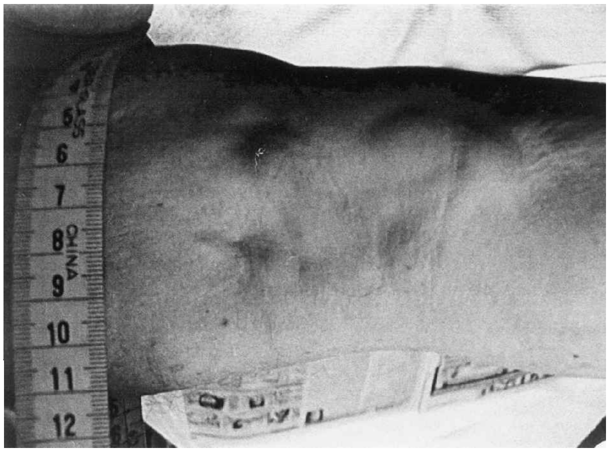

**Fig. 1.** *Macroscopic aspect of the multinodular sclerotic lesion fixed to the skin at the elbow region (forearm is to the right). This appearance remained identical during 16 years after the extravasation and then suddenly changed.*

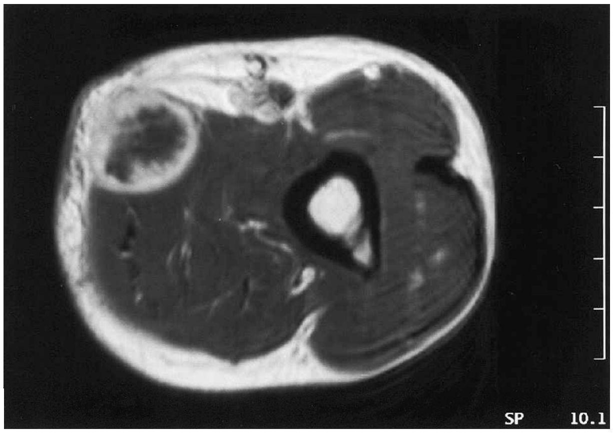

**Fig. 2.** *Transverse section of MRI image just above the elbow. The tumour enhances peripherally with central necrosis.*

struction of the lateral chest wall was carried out with a Vicryl mesh.

Histological examination showed metastases of a high-grade pleomorphic sarcoma in all of the excised tissues.

Because of the bad hematological tolerance of the ifosfamide therapy no further adjuvant therapy was given. The patient remains in close follow-up.

### *Histology*

The tumour at the left elbow was received fresh. A resection specimen consisted of a multinodular whitish and firm, well-delineated tumour. A small tissue fragment was taken for cytogenetic analysis. The remaining tissue was processed to paraffin. On histology, a keloïdal scar replaced the dermal and subcutaneous tissue (Fig. 3). In the deeper part of the scar, a pleomorphic mesenchymal tumour was seen, consisting of plump to spindled tumour cells. The cellularity varied considerably and the matrix ranged from myxoid to collagenous. A sprinkling of inflammatory cells was focally seen. Numerous (a)typical mitoses were present (Fig. 4) and in the centre of the lesion, areas of tumour necrosis were found. Immunohistochemistry, using antibodies against desmin, $\alpha$-smooth muscle actin, cytokeratin, myogenin, and S100 protein, revealed $\alpha$-smooth muscle actin expression in the majority of tumour cells, and a rare desmin-positive cell. Based on these findings, a diagnosis of high-grade pleomorphic sarcoma with some myogenic differentiation was reached.

### *Karyotype of the tumour*

Chromosome metaphases were obtained from 5-day-old cultures of an overnight collagenase disaggregated specimen of the fresh tumour biopsy, utilising standard procedures. G-banded chromosomes were evaluated and classified according to the International System of Human Cytogenetic Nomenclature (1995). Seven metaphases exhibited abnormal karyotype: 44–45,

XY, add(1) (p11), add(4) (q35), -5, dic(7;15) (q11;p11), -8, del(11) (p11), -13, add(13) (p11), -14, -20, +3 r, +1–2 mar [6]/90, idemx2[1]. The remaining 13 cells had a normal male karyotype.

### Discussion

Many epidemiological factors have been suggested in the etiology of STS such as irradiation, chemical carcinogens, genetic disorders, trauma, immunodepression, viruses or even late age at first pregnancy or high body mass index.[1–7] Some of these are well recognised; others are only recognised in experimental animal models.

Adriamycin (doxorubicin hydrochloride) is a widely used anthracycline antibiotic drug especially prone to cause severe tissue damage on extravasation. Accidental extravasation has been estimated to occur in 0.01–6% of all patients receiving chemotherapy.[8–10]

As a topoisomerase II poison, it acts on the so-called cleavable complex in the catalytic cycle of the essential nuclear enzyme topoisomerase II, thereby prolonging the transient stage where the enzyme has locked the gate DNA molecule with a strand break. The drug action becomes lethal because of the accumulation of DNA strand breaks.[8] In addition, the cytotoxic activity of dosorobucin is also related to its quinone structure, which leads to the formation of cytotoxic free radical oxygen intermediates.[11]

The chronicity of doxorubicin extravasation injuries and ulcers is felt to be secondary to its effect on host DNA, resulting in host DNA–doxorubicin complexes, which are released from dead cells and captured by living cells via endocytosis, thereby recycling the necrosing process up to 5 months after extravasation.[9,12–14]

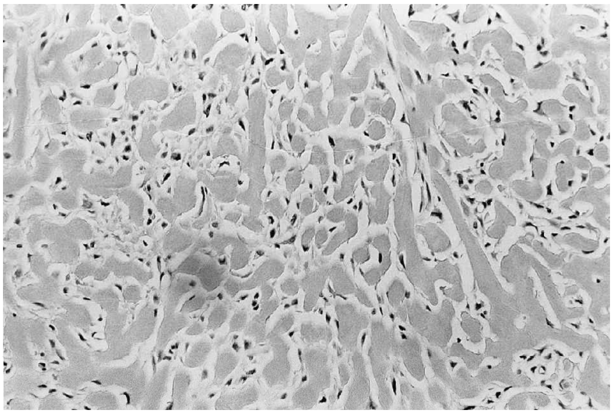

**Fig. 3.** *H&E stain of the keloid scar tissue in the neighbourhood of the sarcoma.*

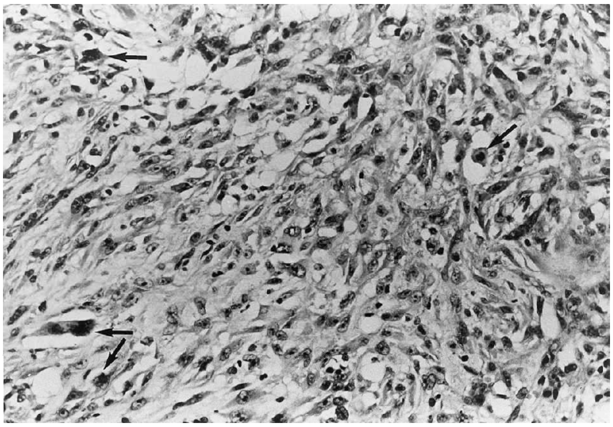

**Fig. 4.** *Hematoxylin and eosin stain, showing a cellular tumour, consisting of plump/spindled tumour cells. There is some inflammatory background. Arrows indicate mitotic figures.*

Histopathological evidence, however, of this prolonged effect is insufficient.

Measurements of doxorubicin levels in human skin 28 days after extravasation showed surprisingly high levels without the presence of any metabolites of doxorubicin in the samples. Histopathological analysis performed on the excised skin showed complete epidermal and subcutaneous necrosis, and reactive hyperplasia and fibrosis.[12]

Examination of unstained frozen sections of punch biopsies taken from a doxorubicin-induced ulcer in a rabbit model by Luedke et al. showed red fluorescence in the nuclei of all cellular elements, indicating persistence of doxorubicin in the tissues 24 and 168 h after the injection. Further histological studies of these doxorubicin-induced ulcers showed a necrotic lesion with a lack of inflammatory response, and a primary vascular or vasospastic mechanism was suggested.[15] This lack of inflammatory infiltrate could be the main factor interfering with revascularisation and healing.[12]

Histological studies of human ulcers 2–3 months after extravasation showed patent blood vessels at the edge of the ulceration and dense scarring in the dermis. As in the observations of Luedke, inflammatory cells were relatively sparse.[16]

Rudolph *et al.* found a normal myofibroblast ultrastructure in experimental skin lesions, induced by intradermal doxorubicin injection in rats, suggesting that the observed lack of wound contraction might be due to cellular dysfunction related to DNA base binding, which would interfere with the cell replication necessary for wound healing.[17]

In the 1960s, Carter *et al.* reported on the early development of injection-site sarcomas in rats.[18–20] They studied the histopathological changes occurring after subcutaneous and intraperitoneal injection of polymerised nitroso-rubber compound[18] and after intramuscular injection of iron-dextran.[19]

Three stages of development were tentatively defined. Tiny foci of abnormal fibroblasts associated with increased amounts of acid mucopolysaccharide ground substance were seen between the pre-existing multinucleate giant cells at the injection sites. These foci enlarged, became progressively cellular and pleomorphic, and formed circumscribed nodules in the second stage. Later these nodules became irregular in outline, invaded adjacent tissues and became recognisable as neoplasms: mainly spindle cell lesions or mixed tumours with pleomorphic elements.

More recently Richter *et al.* studied presarcomatous lesions of experimentally induced sarcomas in rats.[21] A benzopyrene–oil mixture was injected intramuscularly in the thighs of rats. Groups of animals were sacrificed every 10 days. It was found that these injections produced soft tissue sarcomas in a triphasic, overlapping or co-existing pattern that resembled non-healing granulation process.

An initial, acute inflammatory reaction was characterised by an infiltration of lymphocytes, monocytes and macrophages. A second, mesenchymal fibromatous phase was characterised by the predominance of spindle-shaped, focally atypical fibroblast-like cells and collagen. A third, premalignant neovascularisation phase was characterised by dominant capillary proliferation that evolved into overt malignant fibrous histiocytoma (MFH) with pleiomorphic histiocyte- and fibroblast-like cells with atypical mitoses. Massive capillary proliferation preceded the development of manifest sarcomas, indicating angiogenesis as a potential factor in tumour growth.

Kirkpatrick *et al.*[22] studied the development of mesenchymal malignancies after the implantation of eight different biomaterials in a rat model.

Animals were under observation for 8 or 24 months after implantation and were sacrificed for histological and immunohistochemical analysis. Depending on the implanted biomaterial, 12–35% of the implantation sites showed development of one or more sarcomas. The most frequent tumour was malignant fibrous histiocytoma, followed by pleomorphic sarcoma. The authors proposed a multistage tumorigenesis hypothesis, analogous to the well-known adenoma–carcinoma sequence. Histopathological examination of the capsules revealed a variety of morphological entites, which were clearly distinguishable from each other, beginning with paucicellular fibrous tissue tumour. Later, proliferative lesions consisting of groups of polygonal and/or spindle cells were seen. In many cases, foci of proliferation were observed with marked cellular typia such as cellular and nuclear pleomorphism coupled with hyperchromasia and well-defined nucleoli.

A few capsules revealed a further stage in the development of a sarcoma and showed a larger area of markedly atypical cells with an organoid pattern that could be clearly identified as malignant. Certain cases presented a combination of lesions such as manifested sarcoma with a preneoplastic lesion.

This model, however, is limited to signals that are elicited locally. The role of systemic factors in the induction process remains completely unknown, as does the differentiation between local, chemical and physical characteristics as the predominant pathogenic factor.

Postvaccinal sarcoma in cats has been a well-known entity in veterinary medicine for many years. Hendrick *et al.* studied the occurrence of injection-site sarcomas in cats after vaccination with a mandatory rabies vaccine with aluminium-based adjuvants.[23]

Fifty-one percent of the tumours reviewed were surrounded, and partially infiltrated by lymphocytes and macrophages containing aluminium and oxygen as shown by electron probe X-ray microanalysis. The authors suggested that the persistence of the inflammatory and immunological reactions associated with the presence of aluminium in the injection sites predisposed the cat to a disturbance of its fibrous connective tissue repair response, eventually leading to neoplasia in some of the cases. In favour of this hypothesis was the fact that few cases were found to be 'transitional': microscopic foci of sarcoma were found in areas of granulomatous infiltration.

We suggest that the extravasation of doxorubicin in our patient caused a chronic lesion formed due to the long persistence of doxorubicin–DNA complexes in the subcutaneous tissues.

These persistent complexes probably caused a non-specific cellular damage at the nuclear level, interfering with cell replication necessary for normal healing tissues. As a result, this non-healing chronic inflammatory tissue developed into a preneoplastic mesenchymal tumour and many years later into a high-grade pleomorphic sarcoma.

To our knowledge, the case of a STS arising at the site of extravasation of doxorubicin in humans that we present is the only one reported in the literature. It illustrates that, as in experimental animal models, chronic fibrous inflammatory tissue might predispose to sarcoma development. Adequate initial treatment of the extravasation on prophylactic resection of the persistent fibrous tissue might have prevented the sarcoma.

### References

1 Das Gupta TK. *Tumours of the soft tissue*. New York: Appleton & Lange, 1998.

2 Dirix LY, Vermeulen P, De Wever I, Van Oosterom AT. Soft tissue sarcoma in adults. *Curr Opin Oncol* 1997; 9: 348–59.

3 Enzinger F, Weiss S. *Soft tissue tumours*. St. Louis, Mosby Yearbook, 1995.

4 Schwarz RE, Burt M. Radiation-associated malignant tumors of the chest wall. *Ann Surg Oncol* 1996; 3: 387–92.

5 Fizazi K, Cojean I, Le Cesne A, Kayitalire L, Le Chevalier T, Tursz T, Spielmann M. Sarcomes des tissues mous: revue generale. *Bull Cancer* 1994; 81: 835–52.

6 Fioretti F, Tavani A, Gallus S, Negri E, Franceschi S, La Vecchia C. Menstrual and reproductive factors and risk of soft tissue sarcomas. *Cancer* 2000: 4; 786–9.

7 Tavani A, Soler M, La Veccia C, Negri E, Gallus S, Franceschi S. Body weight and risk of soft-tissue sarcoma. *Br J Cancer* 1999: 81; 890–2.

8 Langer SW, Sehested M, Jensen PB. Treatment of anthracycline extravasation with dexrazoxane. *Clin Cancer Res* 2001; 6: 3680–6.

9 Heckler FR. Current thoughts on extravasation injuries. *Clin Plast Surg* 1989; 16: 557–63.

10 Heitmann C, Durmus C, Ingianni G. Surgical management after doxorubicin and epirubicin extravasation. *J Hand Surg (Br)* 1998; 23B 5: 666–8.

11 Monstrey SJ, Mullick P, Narayanan K, Ramasastry SS. Hyperbaric oxygen therapy and free radical production: an experimental study in Doxorubicin (Adriamycin) extravasation injuries. *Ann Plast Surg* 1997; 38: 163–8.

12 Emiroglu M, Erçöçen AR, Demirseren ME, Yenidüna S, Edali N. Histopathological changes in adriamycin extravasation injury. *Ann Plast Surg* 1998; 4: 103–4.

13 Sonneveld P, Wassenaar HA, Nooter K. Long persistence of Doxorubicin in human skin after extravasation. *Cancer Treat Rep* 1984; 68: 895–6.

14 Zweig JI, Kabakow B, Wallach RC, Valenic M, Zalusky R. Rational effective treatment of skin ulcers due to Adriamycin. *Cancer Treat Rep* 1979; 63: 2101–3.

15 Luedke DW, Kennedy PS, Rietschel RL. Histopathogenesis of skin and subcutaneous injury induced by Adriamycin. *Plast Reconstr Surg* 1979; 63 (4): 463–5.

16 Rudolph R, Larson DL. Etiology and treatment of chemotherapeutic agent extravasation injuries: a review. *J Clin Oncol* 1987; 5: 1116–26.

17 Rudolph R, Woodward M, Hurn I. Ultrastructure of doxorubicin (Adriamycin)–induced skin ulcers in rats. *Cancer Res* 1979; 39: 3689–93.

18 Carter RL. Early development of injection-site sarcomas in rats: a study of tumours induced by a rubber additive. *Br J Cancer* 1968; 23 (2): 408–16.

19 Carter RL. Early development of injection-site sarcomas in rats: a study of tumours induced by iron-dextran. *Br J Cancer* 1969; 23 (3): 559–6.

20 Carter RL, Birbeck MSC, Roberts JDB. Development of injection-site sarcomata in rats: a study of the early reactive changes evoked by a carcinogenic nitroso-quinoline compound. *Br J Cancer* 1970; 24(2): 300–11.

21 Richter KK, Parham DM, Scheele J, Hinze R, Rath FW. Presarcomatous lesions of experimentally induced sarcomas in rats: morphologic, histochemical and immunohistochemical features. *In Vivo* 1999; 13: 349–56.

22 Kirkpatrick CJ, Alves A, Köhler H, Kriegsmann J, Bittinger F, Otto M, Williams DF, Eloy R. Biomaterial-induced sarcoma. *Am J Pathol* 2000; 4: 1455–67.

23 Hendrick MJ, Goldschmidt MH, Shofer FS, Wang Y, Somlyo A. Postvaccinal sarcomas in the cat: epidemiology and electron probe microanalytical identification of aluminium. *Cancer Res* 1992; 52: 5391–4.

24 Bertelli G. Prevention and management of extravasation of cytotoxic drugs. *Drug Saf* 1995; 12: 245–55.
25 Larson DL. What is the appropriate management of tissue extravasation by antitumour agents? *Plast Reconstr Surg* 1985; 75: 397–402.
26 Disa JJ, Chang RR, Mucci SJ, Goldberg NH. Prevention of Adriamycin-induced full-thickness skin loss using hyaluronidase infiltration. *Plast Reconstr Surg* 1998; 101: 370–4.
